# Supplementary material for: Effects of Dickkopf-1 (DKK-1) on Prostate Cancer Growth and Bone Metastasis
Source: Cells. 2023 Nov 24;12(23):2695. doi: 10.3390/cells12232695 (PMC10705757; doi:10.3390/cells12232695)
Supplement: Supplementary file 1 [file cells-12-02695-s001.zip › cells-2687188-supplementary.pdf]

Supplementary figures

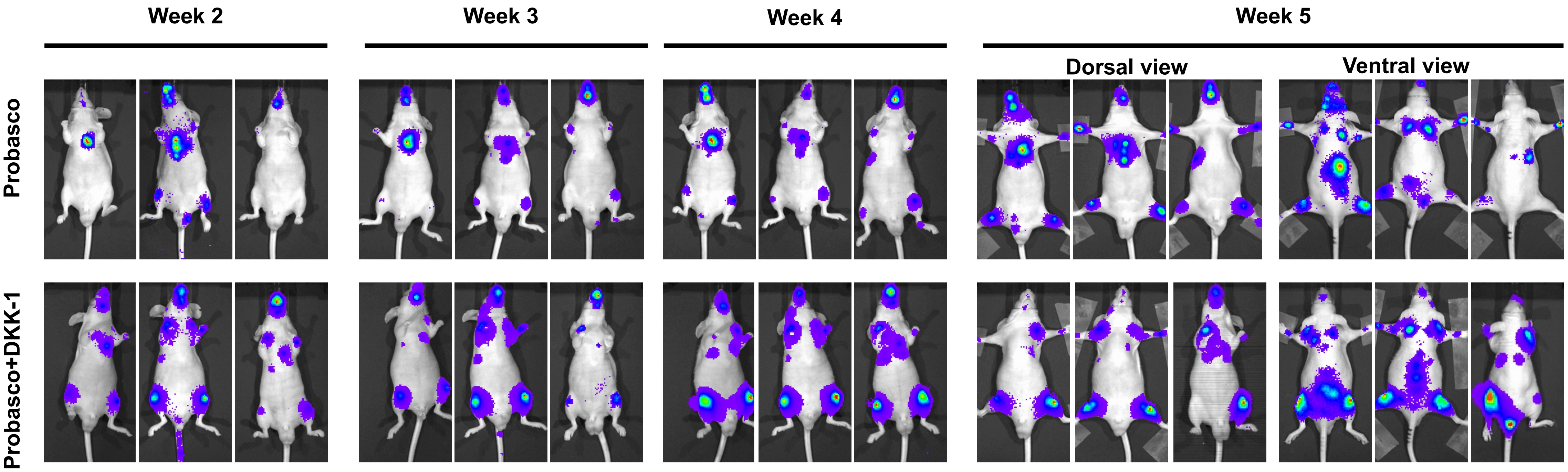

Figure S1: Representative bioluminescent images of intracardiac injected nude mice. Metastasis and growth of cancer cells were monitored by bioluminescent imaging weekly after injections. Mice were euthanized at week 5 (signals detected from the chest region were due to the growth of cancer cells that leaked during intracardiac injections and were not counted as metastatic sites).

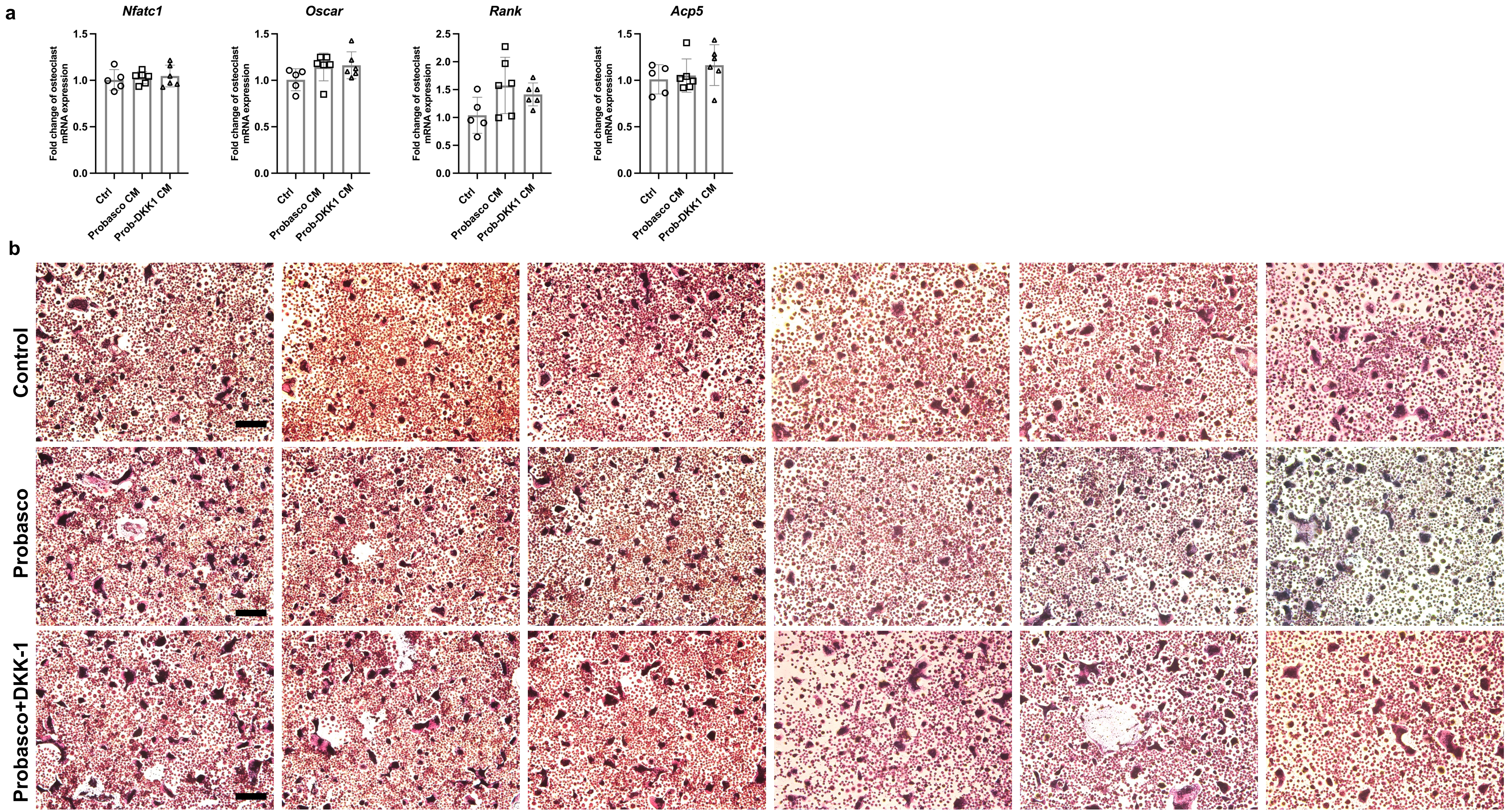

Figure S2: Effects of Probasco or Probasco+DKK-1 conditioned medium (CM) on primary murine osteoclasts. (a) mRNA expression of additional osteoclasts markers, *Nfatc1*, *Oscar*, *Rank*, and *Acp5,* in control (ctrl) and CM-treated osteoclasts. (b) images of TRAP-stained osteoclasts from all replicates. n=5 to 6 for each osteoclast group. Bar=200µm. Data was displayed as mean±SD.

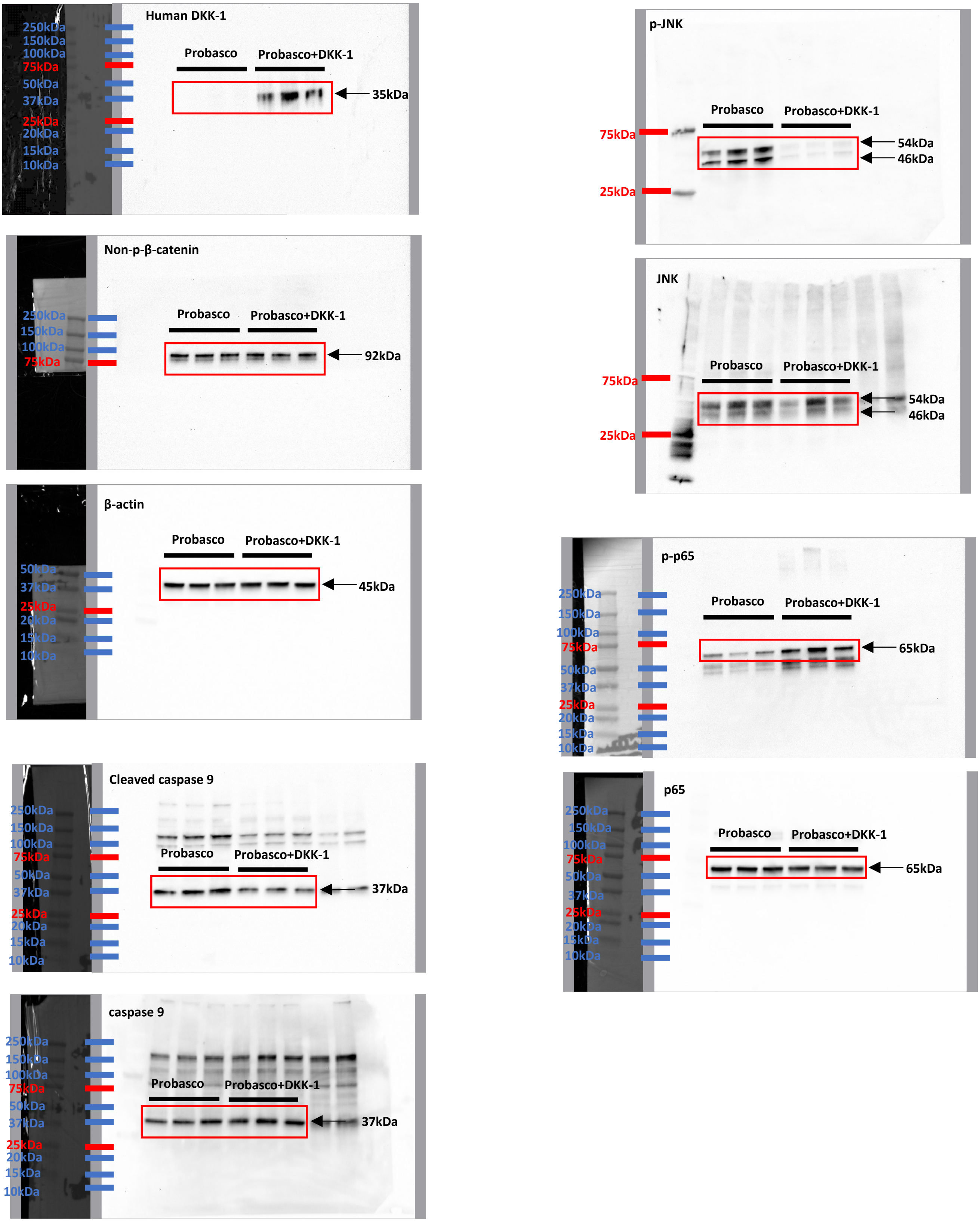

Figure S3: Original western blot images for Figure 6.
